# SOCIAL AND BEHAVIORAL FACTORS IN COGNITIVE AGING: APPLYING THE CAUSAL INFERENCE FRAMEWORK IN OBSERVATIONAL STUDIES

**DOI:** 10.1093/geroni/igz038.1178

**Published:** 2019-11-08

**Authors:** Anja K Leist

**Affiliations:** University of Luxembourg, Esch-sur-Alzette, Luxembourg

## Abstract

Rationale: There is an urgent need to better understand how to maintain cognitive functioning at older ages with social and behavioral interventions, given that there is currently no medical cure available to prevent, halt or reverse the progression of cognitive decline and dementia. However, in current models, it is still not well established which factors (e.g. education, BMI, physical activity, sleep, depression) matter most at which ages, and which behavioral profiles are most protective against cognitive decline. In the last years, advances in the fields of causal inference and machine learning have equipped epidemiology and social sciences with methods and models to approach causal questions in observational studies. Method: The presentation will give an overview of the causal inference framework and different machine learning approaches to investigate cognitive aging. First, we will present relevant research questions on the role of social and behavioral factors in cognitive aging in observational studies. Second, we will introduce the causal inference framework and recent methods to visualize and compute the strength of causal paths. Third, promising machine learning approaches to arrive at robust predictions are presented. The 13-year follow-up from the European SHARE survey that employs well-established cognitive performance tests is used to demonstrate the usefulness of the approach. Discussion: The causal inference framework, combined with recent machine learning approaches and applied in observational studies, provides a robust alternative to intervention research. Advantages for investigations under the new framework, e.g., fewer ethical considerations compared to intervention research, as well as limitations are discussed.

